# Ribociclib-Induced Autoimmune Hepatitis: A Case Report

**DOI:** 10.7759/cureus.71817

**Published:** 2024-10-18

**Authors:** Beyza Atay, Ali Berkcan Bozdogan, Yigit Yazarkan, Muhammed Bahaddin Durak

**Affiliations:** 1 Department of Internal Medicine, Division of Gastroenterology, Hacettepe University, Ankara, TUR; 2 Department of Internal Medicine, Hacettepe University, Ankara, TUR

**Keywords:** auto-immune hepatitis, cdk 4/6 inhibitor, drug induced hepatitis, drug-induced liver injury (dili), ribociclib

## Abstract

Drug-induced autoimmune hepatitis (DI-AIH) is a condition that mimics autoimmune hepatitis both histologically and clinically, making diagnosis challenging. Ribociclib, a CDK4/6 inhibitor used in the treatment of metastatic breast carcinoma, has been associated with rare cases of DI-AIH. We present the case of a 46-year-old woman undergoing treatment with ribociclib for metastatic breast carcinoma, who developed fatigue, skin rash, and significantly elevated liver enzymes two months into therapy. Initial tests revealed ALT of 414 U/L and AST of 219 U/L, along with elevated IgG levels and positive antinuclear antibodies. A liver biopsy showed chronic active hepatitis with mixed inflammatory cell infiltrates, including plasma cells and eosinophils. Despite the discontinuation of ribociclib, the liver enzymes remained elevated, necessitating treatment with prednisolone and azathioprine. Over the course of six months, the patient’s liver function improved, and immunosuppressive therapy was gradually tapered off. This case highlights the importance of recognizing ribociclib-induced DI-AIH and the effectiveness of immunosuppressive therapy in managing persistent liver injury.

## Introduction

Autoimmune hepatitis (AIH) is a chronic liver disorder characterized by interface hepatitis, resulting from inflammatory processes. The precise etiology of the condition remains unidentified. Due to the high recurrence rate after immunosuppressive therapy, lifelong treatment may often be required [1]. It is well-established that drug-induced liver injury (DILI) may mimic both acute and chronic liver diseases [2]. Drug-induced autoimmune hepatitis (DI-AIH) is defined as drug-induced liver injury with laboratory and/or histological features indistinguishable from AIH [3]. It remains challenging to distinguish between AIH and DILI, despite some reports suggesting that AIH can be differentiated by significant eosinophilic infiltrates, increased portal neutrophils, and cholestasis. Recently, ribociclib, a cyclin-dependent kinase 4 and 6 (CDK4/6) inhibitor commonly used to treat metastatic breast cancer, has been increasingly linked to DILI. Ribociclib induced-liver injury mechanism was linked to DI-AIH in extremely rare cases. Here, we aimed to discuss a case of ribociclib-induced DI-AIH.

## Case presentation

**Figure 1:**
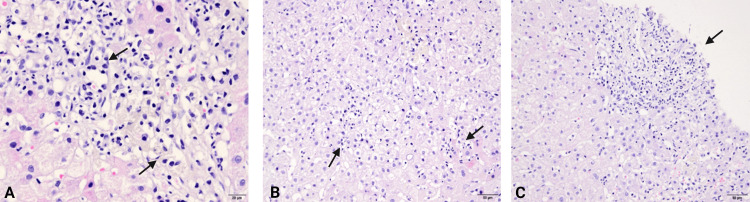
Liver biopsy findings. A. Portal area with patent bile duct, portal vein, and artery, accompanied by mixed-type inflammation consisting of lymphocytes, plasma cells, and few eosinophils. Focal irregularity at the portoparenchymal border due to inflammation. B. Necrotic, inflammatory foci observed scattered throughout the liver parenchyma. C. Mixed-type inflammation consisting of lymphocytes, plasma cells, and few eosinophils.

A 46-year-old woman presented with fatigue, skin rash, and elevated liver enzymes two months after starting ribociclib treatment for metastatic breast carcinoma (stage IV). Alanine aminotransferase (ALT) was 414 U/L, aspartate aminotransferase (AST) was 219 U/L, alkaline phosphatase (ALP) was 72 U/L, gamma-glutamyl transferase (GGT) was 94 U/L, total bilirubin was 1.40 mg/dL, direct bilirubin was 0.35 mg/dL, and lactate dehydrogenase (LDH) was 327 U/L, despite liver enzyme levels being within normal limits before treatment. The patient had no history of alcohol consumption or use of other possible hepatotoxic agents, and viral serological tests were negative. Serum IgG level was 2450 mg/dL, and antinuclear antibody titer was >1/160. Ultrasonography showed no evidence of chronic liver disease. A liver biopsy was performed on the 10th day following drug discontinuation due to the persistence of liver enzyme elevation and a score of 12 (possible AIH) for AIH diagnostic criteria from the revised original scoring system of the International Autoimmune Hepatitis Group. Liver biopsy showed mixed-type inflammation with lymphocytes, plasma cells, and few eosinophils in the portal area and moderate interface hepatitis (Figure 1).

**Figure 2:**
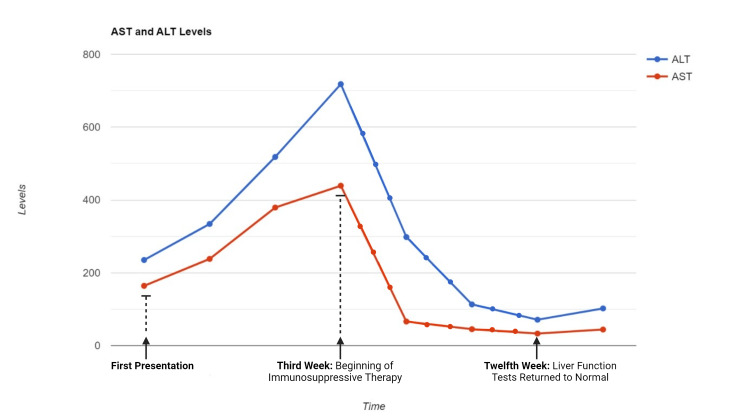
Course of ALT and AST levels

The viral hepatitis panel, anti-LKM1, and SMA were within normal limits. Abdominal ultrasound was inconclusive. The liver biopsy findings indicated chronic active hepatocellular injury with a propensity for chronicity, which did not exhibit typical features of autoimmune hepatitis but could not be ruled out. The Hepatic Activity Index was 8 out of 16, and fibrosis was graded 1 out of 6. Glucocorticoid therapy (prednisolone, 40 mg) together with azathioprine (50 mg) was initiated. After six months of therapy with glucocorticoids and seven months with azathioprine, subsequent follow-ups showed a significant improvement in liver function tests. Prednisolone 40mg/day p/o and azathioprine 50mg/day combination was given. Following the treatment, liver enzymes tended to decrease at the end of the first week and were at normal levels in the third month (Figure 2). After the liver enzymes improved and returned to normal levels, the immunosuppressive dose was reduced and discontinued in the sixth month. During the immunosuppressive therapy period, the patient received radiotherapy and letrozole treatment. After the discontinuation of immunosuppressive therapy, ribociclib treatment was initiated again. No side effects have been seen to date.

## Discussion

Ribociclib is an orally administered, highly selective reversible inhibitor of CDK4 and CDK6, endorsed by the Food and Drug Administration (FDA) for the treatment of hormone receptor-positive (HR+) metastatic breast cancer when used in conjunction with specific endocrine therapies [4]. The toxicity characteristics of CDK4/6 inhibitors have been described in the MONALEESA clinical trial [5]. Ribociclib can lead to liver toxicity, necessitating the monitoring of liver function tests before and during treatment. According to the drug's label instructions, liver function tests should be conducted prior to initiating therapy, at two-week intervals during the first two cycles, at the start of the next four cycles, and as clinically indicated thereafter to ensure patient safety. Management of ribociclib-induced hepatotoxicity may require dose adjustments or discontinuation [4,6]. Previous research has demonstrated that individuals diagnosed with DI-AIH and those with AIH share remarkably similar clinical, biochemical, immunological, and histological characteristics [3,7]. Patients with AIH typically need long-term immunosuppression, in contrast to DI-AIH, in which patients often only need short-term medication and relapse is unlikely following drug withdrawal. Due to the difference in therapy duration, it is crucial to distinguish between the two conditions [1,3]. DI-AIH often presents acutely, with IgM subclass autoantibodies that decline after drug discontinuation, whereas idiopathic AIH involves both IgM and IgG autoantibodies that persist and require long-term immunosuppression. Histologically, advanced fibrosis is more common in idiopathic AIH, while DI-AIH resolves after stopping the causative drug [8]. Nitrofurantoin, methyldopa, hydralazine, minocycline, infliximab, as well as more than 1000 different drugs including herbal and dietary supplements have been shown to play a role in DI-AIH [9]. Although liver damage associated with CDK 4/6 inhibitors has been reported, the mechanism of injury attributed to DI-AIH is not well defined [7]. Further studies are needed to clarify the link between ribociclib and AIH. The present case is a presentation of ribociclib-induced AIH.

Reported cases have demonstrated patterns of hepatocellular liver injury, characterized by a sustained increase in transaminase levels even after the immediate discontinuation of ribociclib following elevating liver enzymes [10,11]. A broad range of mixed patterns of liver injury has been noted, ranging from necrosis to fulminant hepatitis with no necrosis. These cases usually responded to steroid treatment [12]. In our case, despite ribociclib discontinuation, liver enzyme elevation persisted, so azathioprine treatment combined with steroids was administered, and the treatment was terminated in the sixth month following improvement in liver enzymes.

## Conclusions

In this case study, we present a rare instance of ribociclib-induced DI-AIH in a patient undergoing treatment for metastatic breast carcinoma. The clinical presentation, including elevated liver enzymes and histological findings of mixed-type inflammation, aligns with DI-AIH despite the absence of typical autoimmune hepatitis features. The patient's response to a combination of glucocorticoids and azathioprine underscores the potential for effective management of DI-AIH, though distinguishing it from true autoimmune hepatitis remains challenging. With the rising use of CDK4/6 inhibitors like ribociclib, it is crucial to emphasize the importance of vigilant liver function monitoring to promptly identify and manage potential hepatic complications. Future research is needed to further elucidate the mechanisms linking ribociclib to autoimmune liver injury and to refine management strategies for such drug-induced conditions.
